# Online Phylogenetics with matOptimize Produces Equivalent Trees and is Dramatically More Efficient for Large SARS-CoV-2 Phylogenies than *de novo* and Maximum-Likelihood Implementations

**DOI:** 10.1093/sysbio/syad031

**Published:** 2023-05-26

**Authors:** Alexander M Kramer, Bryan Thornlow, Cheng Ye, Nicola De Maio, Jakob McBroome, Angie S Hinrichs, Robert Lanfear, Yatish Turakhia, Russell Corbett-Detig

**Affiliations:** Department of Biomolecular Engineering, University of California Santa Cruz, Santa Cruz, CA 95064, USA; Genomics Institute, University of California Santa Cruz, Santa Cruz, CA 95064, USA; Department of Biomolecular Engineering, University of California Santa Cruz, Santa Cruz, CA 95064, USA; Genomics Institute, University of California Santa Cruz, Santa Cruz, CA 95064, USA; Department of Electrical and Computer Engineering, University of California San Diego, San Diego, CA 92093, USA; European Molecular Biology Laboratory, European Bioinformatics Institute (EMBL-EBI), Wellcome Genome Campus, Cambridge CB10 1SD, UK; Department of Biomolecular Engineering, University of California Santa Cruz, Santa Cruz, CA 95064, USA; Genomics Institute, University of California Santa Cruz, Santa Cruz, CA 95064, USA; Genomics Institute, University of California Santa Cruz, Santa Cruz, CA 95064, USA; Department of Ecology and Evolution, Research School of Biology, Australian National University, Canberra, ACT 2601, Australia; Department of Electrical and Computer Engineering, University of California San Diego, San Diego, CA 92093, USA; Department of Biomolecular Engineering, University of California Santa Cruz, Santa Cruz, CA 95064, USA; Genomics Institute, University of California Santa Cruz, Santa Cruz, CA 95064, USA

**Keywords:** SARS-CoV-2, phylogenetics, parsimony, maximum likelihood, optimization

## Abstract

Phylogenetics has been foundational to SARS-CoV-2 research and public health policy, assisting in genomic surveillance, contact tracing, and assessing emergence and spread of new variants. However, phylogenetic analyses of SARS-CoV-2 have often relied on tools designed for *de novo* phylogenetic inference, in which all data are collected before any analysis is performed and the phylogeny is inferred once from scratch. SARS-CoV-2 data sets do not fit this mold. There are currently over 14 million sequenced SARS-CoV-2 genomes in online databases, with tens of thousands of new genomes added every day. Continuous data collection, combined with the public health relevance of SARS-CoV-2, invites an “online” approach to phylogenetics, in which new samples are added to existing phylogenetic trees every day. The extremely dense sampling of SARS-CoV-2 genomes also invites a comparison between likelihood and parsimony approaches to phylogenetic inference. Maximum likelihood (ML) and pseudo-ML methods may be more accurate when there are multiple changes at a single site on a single branch, but this accuracy comes at a large computational cost, and the dense sampling of SARS-CoV-2 genomes means that these instances will be extremely rare because each internal branch is expected to be extremely short. Therefore, it may be that approaches based on maximum parsimony (MP) are sufficiently accurate for reconstructing phylogenies of SARS-CoV-2, and their simplicity means that they can be applied to much larger data sets. Here, we evaluate the performance of *de novo* and online phylogenetic approaches, as well as ML, pseudo-ML, and MP frameworks for inferring large and dense SARS-CoV-2 phylogenies. Overall, we find that online phylogenetics produces similar phylogenetic trees to *de novo* analyses for SARS-CoV-2, and that MP optimization with UShER and matOptimize produces equivalent SARS-CoV-2 phylogenies to some of the most popular ML and pseudo-ML inference tools. MP optimization with UShER and matOptimize is thousands of times faster than presently available implementations of ML and online phylogenetics is faster than *de novo* inference. Our results therefore suggest that parsimony-based methods like UShER and matOptimize represent an accurate and more practical alternative to established ML implementations for large SARS-CoV-2 phylogenies and could be successfully applied to other similar data sets with particularly dense sampling and short branch lengths.

The widespread availability and extreme abundance of pathogen genome sequencing has made phylogenetics central to combatting the COVID-19 pandemic. Communities worldwide have implemented genomic surveillance by systematically sequencing the genomes of a percentage of local cases (Deng et al. 2020; Lu et al. 2020a; Meredith et al. 2020; Park et al. 2021). This has been important in tracing local transmission chains (Bluhm et al. 2020; Lam 2020), understanding the genetic makeup of viral populations within local communities (Gonzalez-Reiche et al. 2020; Franceschi et al. 2021; Thornlow et al. 2021a), uncovering the means by which viral lineages have been introduced to new areas (Castillo et al. 2020), and measuring the relative spread of specific variants (Skidmore et al. 2021; Umair et al. 2021). Phylogenetic approaches for better understanding the proximate evolutionary origins of the virus (Li et al. 2020), as well as to identify recombination events (Jackson et al. 2021; Turakhia et al. 2021b) and instances of convergent evolution (Kalantar et al. 2020; Peng et al. 2021) have greatly informed our understanding of the virus. Phylogenetic visualization software including Auspice (Hadfield et al. 2018) and Taxonium (Sanderson 2022) have also become widely used for public health purposes. All of these applications require a phylogeny.

A comprehensive, up-to-date phylogenetic tree of SARS-CoV-2 is important for public health officials and researchers. A tree containing all available sequences can sometimes facilitate identification of epidemiological links between samples that might otherwise be lost in subsampled phylogenies. Conversely, these approaches can often rule out otherwise plausible transmission histories. Such information can also help to identify the likely sources of new viral strains in a given area (Moreno et al. 2020; Tang et al. 2021). Additionally, using up-to-date information enables us to find and track novel variants of concern and clades which are growing quickly (Annavajhala et al. 2021; Tegally et al. 2021), and to measure the spread of known variants at both local and global scales. Furthermore, comprehensive phylogenies can improve the ability of computational methods to find recombinant viral genomes (Turakhia et al. 2021b), natural selection at homoplasious positions (van Dorp et al. 2020), variation in mutation rates (De Maio et al. 2021), and systematic recurrent errors (Turakhia et al. 2020). This also facilitates naming lineages of interest, which has been particularly important in tracking variants of concern throughout the pandemic (*e.g.,* B.1.1.7 or “Alpha” and B.1.617.2 or “Delta”) (Rambaut et al. 2020).

SARS-CoV-2 presents a unique set of challenges for phylogenetic analyses. The unprecedented pace and scale of whole-genome sequence data has forced the phylogenetics community to place runtime and scalability at the center of every inference strategy. More than 14 million SARS-CoV-2 genome sequences are currently available, with tens of thousands being added each day. Prior to the pandemic, *de novo* phylogenetics, that is, inferring a phylogeny from scratch, has been the standard, as there has rarely been a need to re-infer or improve pre-existing phylogenies on a daily basis. Daily inference of a tree of millions of samples from scratch, however, is extremely costly, and has brought a renewed focus on methods for adding new samples to existing phylogenetic trees (Matsen et al. 2010; Berger et al. 2011; Izquierdo-Carrasco et al. 2014; Fourment et al. 2018; Barbera et al. 2019). This approach has been called “online phylogenetics” (Gill et al. 2020), and has important advantages in the context of the pandemic and beyond. Online phylogenetics is appealing for the genomic surveillance of pathogens, because iterative optimization should decrease computational expense, allowing good estimates of phylogenies to be made readily available. It may be particularly effective in a pandemic setting, where new samples are closely related to existing samples in the tree, and existing samples are often identical to inferred ancestral states. That is, the ancestor of a newly sequenced genome has often effectively already been observed. The methods described here may be less useful for reconstructing deeper evolutionary histories of other large genomic data sets, for example organisms like insects or certain pathogens like HIV-1.

Second, SARS-CoV-2 genomes are much more closely related than sequences in most other phylogenetic analyses. Because the relative advantages of maximum likelihood (ML) methods decrease for closely related samples and long branches are relatively rare in the densely sampled SARS-CoV-2 phylogeny (Felsenstein 1978; Hendy and Penny 1989; Philippe et al. 2005), this suggests that phylogenetic inferences based on maximum parsimony, a much faster and simpler phylogenetic inference method, could be better suited for online phylogenetic analyses of SARS-CoV-2 genomes (Wertheim et al. 2022). The principle of maximum parsimony is that the tree with the fewest mutations should be favored, and it is sometimes described as a non-parametric phylogenetic inference method (Sullivan and Swofford 2001; Kolaczkowski and Thornton 2004). Additionally, because parsimony-based tree optimization does not require estimation of ancestral character state uncertainty at all positions in the phylogeny like ML optimization does, parsimony uses much less memory.

Here, we evaluate approaches that would enable one to maintain a fully up-to-date and comprehensive global phylogeny of SARS-CoV-2 genome sequences (McBroome et al. 2021). Specifically, we investigate tradeoffs between online and *de novo* phylogenetics and between maximum parsimony, ML, and pseudo-ML approaches when the aim is for an analysis to scale to millions of sequences, with tens of thousands of new sequences being added daily. We chose to compare maximum parsimony, ML, and pseudo-ML (and omit other approaches like neighbor-joining) because they were the most effective methods at inferring large SARS-CoV-2 phylogenies based on previous analyses (Lanfear and Mansfield 2020), and because most distance-based methods are quadratic in memory usage so cannot scale to estimating trees from data sets of more than a few hundred thousand sequences (Wang et al. 2022). We mimic the time-course of the pandemic by introducing increasingly large numbers of SARS-CoV-2 genome sequences proportionately to their reported sampling dates.

We evaluate potential online phylogenetics approaches by iteratively adding samples to existing trees and optimizing the augmented phylogeny with different tools that have been proposed for this purpose during the pandemic. In particular, we compare online matOptimize, IQ-TREE 2, RAxML-NG, and FastTree 2. Between each optimization step, we use UShER (Turakhia et al. 2021a) to add samples to trees by maximum parsimony. matOptimize is a parsimony optimization approach that uses subtree pruning and regrafting (SPR) moves to minimize the total mutations in the final tree topology (Ye et al. 2022). IQ-TREE 2 uses nearest neighbor interchange (NNI) and optionally stochastic tree moves to find the tree with the highest likelihood given an input multiple sequence alignment (Minh et al. 2020). RAxML-NG is a ML approach that uses SPR moves to search tree-space for higher likelihood phylogenies (Kozlov et al. 2019). FastTree 2 uses a pseudo-likelihood approach that employs minimum-evolution SPR and/or NNI moves and ML NNI moves while using several heuristics to reduce the search space (Price et al. 2010). The likelihood-based approaches evaluated here report branch lengths in substitutions per site. Parsimony-based matOptimize reports branch lengths in total substitutions, which can be converted to the latter by dividing by the alignment length. Reporting unambiguous branch lengths in this way is possible because UShER and matOptimize resolve potential ambiguities in ancestral sequences by parsimony. When there are ambiguous ancestral states, both matOptimize and UShER will prefer to assign new mutations at the node farthest from the root. This is similar to DELTRAN (Agnarsson and Miller 2008; Swofford and Maddison 1987). Additionally, when UShER places new samples with ambiguous nucleotides in the consensus sequence, it resolves each to the most parsimonious state given the guide tree. If there is more than one equally parsimonious nucleotide, UShER will select the reference nucleotide if it is one of the possibilities. This decision is motivated by the observation that infrequent ambiguous states arise for a variety of technical reasons and typically the reference nucleotide will be correct (De Maio et al. 2020; Turakhia et al. 2020).The branch lengths reported by UShER and matOptimize may be interpreted as is or used as an initial estimate for other distance measures, for example in the construction of time trees (Sanderson 2021).

Results from our comparisons demonstrate that for the purposes of SARS-CoV-2 phylogenetics, in which samples are numerous and closely related and inference speed is of high significance, parsimony-based online phylogenetics applications are clearly most favorable and are also the only immediately available methods capable of producing daily phylogenetic estimates of all available SARS-CoV-2 genomes (Turakhia et al. 2021a). We note that matOptimize is used to maintain such a phylogeny comprising over 14 million genomes as of February 2023 (McBroome et al. 2021). As global genomic data collection accelerates further, we expect parsimony-based online approaches to become increasingly central to phylogenetic inference for data sets with similar properties to SARS-CoV-2, for example Mpox, RSV, and Mycobacterium tuberculosis.

## Methods

UShER and matOptimize are available through Anaconda at https://anaconda.org/bioconda/usher and on Github at https://github.com/yatisht/usher. Our analyses use Github commit 66ca5ff which corresponds to the nearest conda version of 0.5.0.

### Constructing an Initial Global SARS-CoV-2 Phylogeny and a “Ground Truth” Tree

We first developed a global phylogeny, hereafter the “starting tree,” which we used in subsequent analyses. We began by downloading VCF and FASTA files corresponding to 18 March 2021 from our own daily updated database (McBroome et al. 2021). The VCF file contains pairwise alignments of each of the 434,063 samples to the SARS-CoV-2 reference genome. We then implemented filters, retaining only sequences containing at least 28,000 non-N nucleotides, and fewer than 2 non-[ACGTN-] characters. We used UShER to create a phylogeny from scratch using only the remaining 366,492 samples. To remove potentially erroneous sequences, we iteratively pruned this tree of highly divergent internal branches with branch parsimony scores greater than 30, then terminal branches with branch parsimony scores greater than 6, until convergence, resulting in a final global phylogeny containing 364,427 samples. The branch parsimony score indicates the total number of substitutions along a branch. Similar filters based on sequence divergence are used by existing SARS-CoV-2 phylogenetic inference methods. For full reproducibility, files used for creating the global phylogeny can be found in subrepository 1 on the project GitHub page (Thornlow et al. 2021b).

Following this, we created a “ground truth” tree by optimizing the starting tree to compare against the results of inference methods on simulated data. We used matOptimize, FastTree 2, and maximum parsimony (MP) IQ-TREE 2. MP IQ-TREE 2 uses parsimony as the optimality criterion in contrast to the ML mode used in other experiments, which was infeasible on a data set of this size. In these optimization experiments, we used experimental versions of MP IQ-TREE 2 that allow finer control of parsimony parameters (specific versions are listed in the supplemental Github repository). In one of our Ground Truth Optimization Experiments, we used the starting tree and its corresponding alignment and ran 5 iterations of MP IQ-TREE 2, varying the SPR radius from 20 to 100 in increments of 20. Experiments on a small data set indicated that there is little or no improvement in parsimony score beyond a radius of 100. Separately, we tested another strategy that applied 2 iterations of MP IQ-TREE 2 to the starting tree, the 1st iteration using an SPR radius of 20 and the 2nd using a radius of 100. Finally, we tested a strategy of 6 iterations of pseudo-likelihood optimization with FastTree 2 followed by 2 iterations of parsimony optimization with matOptimize. The tree produced by this strategy, hereafter the “ground truth” tree, had the highest likelihood of all the strategies we tested. This tree (after_usher_optimized_fasttree_iter6.tree) and files for these optimization experiments can be found in subrepository 2.

In the multifurcating ground truth tree of 364,427 samples, there are 265,289 unique (in FASTA sequence) samples. There are 447,643 nodes in the tree. For reference, a full binary tree with the same number of leaves has 728,853 nodes. 23,437 of the 29,903 sites in the alignment are polymorphic (they display at least 2 non-ambiguous nucleotides). Out of 83,216 inferred ancestral nodes, 68,261 (82%) have as a child a sampled node with identical genotype (branch length zero). Homoplasies are common in these data. In the starting tree, 19,090 sites display a mutation occurring on at least 2 different branches, and 4976 sites display a mutation occurring more than 10 times in the tree. Approximately half of the internal nodes are resolved, having exactly 2 nodes as immediate descendants (Supplementary Fig. S3).

### Analyses Using Simulated SARS-CoV-2 Data Under Pandemic Time Constraints

To generate our simulated data, we used the SARS-CoV-2 reference genome (GISAID ID: EPI_ISL_402125; GenBank ID: MN908947.3) (Shu and McCauley 2017; Sayers et al. 2021) as the root sequence and used phastSim (De Maio et al. 2022) to simulate according to the ground truth phylogeny described above. Intergenic regions were evolved using phastSim using the default neutral mutation rates estimated in ref. (De Maio et al. 2021), with position-specific mean mutation rates sampled from a gamma distribution with alpha = beta = 4, and with 1% of the genome having a 10-fold increase mutation rate for one specific mutation type (SARS-CoV-2 hypermutability model described in ref. [De Maio et al. 2022]). Evolution of coding regions was simulated with the same neutral mutational distribution, with a mean nonsynonymous/synonymous rate ratio of omega = 0.48 as estimated in (Turakhia et al. 2021a), with codon-specific omega values sampled from a gamma distribution with alpha = 0.96 and beta = 2. Rates for each intergenic and coding region were not normalized in order to have the same baseline neutral mutation rate distribution across the genome.

We then tested *de novo* and online matOptimize, ML IQ-TREE 2, and FastTree2 on this simulated alignment. The Simulated Online Experiments began by using UShER to infer a small tree *de novo* from the 1st batch of samples, followed by alternating steps of optimization using 1 of the 3 evaluated methods and placement of additional samples with UShER. For our Simulated *De Novo* Experiments, we supplied each software package with an alignment corresponding to all samples in that batch and its predecessors (or VCF for matOptimize) without a guide tree. For both cases, each tree is larger than its predecessor by ~5000 samples, and each tree necessarily contains all samples in the immediately preceding tree. We terminated experiments that took longer than 24 h to complete to mimic the time constraints of continual inference during a pandemic. For FastTree 2, we used 2 rounds of minimum-evolution subtree-prune-regraft (SPR) moves (-spr 2), maximum SPR length of 1000 (-sprlength 1000), zero rounds of minimum-evolution NNIs (-nni 0), default settings for ML nearest neighbor interchanges, and the Generalised Time Reversible+Gamma (GTR+G) substitution model (-gtr -gamma). Previous analyses with FastTree 2 on large SARS-CoV-2 data sets show that an SPR radius of 1000 has negligible effect on execution time but can produce more optimal phylogenies than the default setting of 10 (Lanfear and Mansfield 2020). For IQ-TREE 2, we used a branch length minimum of 0.000000001 (-blmin 1e-9), zero rounds of stochastic tree search (-n 1), and the GTR+G substitution model (-m GTR+G). With these parameters, IQ-TREE 2 constructs a starting parsimony tree and then performs hill-climbing NNI steps to optimize likelihood, avoiding the significant time overhead of stochastic search. We ran matOptimize and UShER with default parameters. For each iteration of our Simulated *De Novo* Experiment with UShER+matOptimize, we started with an empty tree, added all samples with UShER, then optimized with matOptimize. We ran all matOptimize analyses using an instance with 15 CPUs and 117.2 GB of RAM, and we ran all IQ-TREE 2 and FastTree 2 analyses on an instance with 31 CPUs and 244.1 GB of RAM, but we limited each command to 15 threads for equivalence with matOptimize.

To assess the effectiveness of each method, we computed the Robinson–Foulds (RF) distance (Robinson and Foulds 1981) and quartet similarity of each optimization to the ground truth tree, pruned to contain only the samples belonging to that batch. To calculate each RF distance, we used the -O (collapse tree) argument in matUtils extract (McBroome et al. 2021) and then used the dist.topo command in the *ape* package in R (Paradis and Schliep 2019), comparing the collapsed optimized tree and the pruned, collapsed ground truth tree at each iteration. We computed normalized RF distances by dividing each distance by the expression I(Ti) + Ni − 3. The value I(Ti) is the number of internal edges in the pruned ground truth tree for iteration i, and Ni is the number of taxa, with Ni − 3 representing the maximum possible number of internal edges in the inferred tree (Steel and Penny 1993). We computed normalized quartet similarities to the ground truth tree using the metric described in (Asher and Smith 2022) which does not penalize excess resolution in the inferred tree. Files for all simulated data experiments can be found in subrepository 4.

### Analyses Using Real SARS-CoV-2 Data Under Pandemic Time Constraints

To mimic pandemic-style phylogenetics, we separated a total of 233,326 real SARS-CoV-2 samples from the starting tree of 364,427 samples into 50 batches of ~5000 by sorting according to the date of sample collection. We then set up 2 experiments for each of the 3 software packages (matOptimize [conda version 0.5.0], ML IQ-TREE 2 [multicore version 2.1.3 COVID-edition], and FastTree 2 [Double Precision version 2.1.10]). We repeated our iterative experiments using online and *de novo* matOptimize, IQ-TREE 2 and FastTree 2 on real data, using the same strategies as with simulated data, terminating experiments that took more than 24 h. In these Real Online and *De Novo* Experiments, we measured the Jukes-Cantor (JC) likelihood of each tree, as well as the runtime and peak memory usage of each program. Files for all real data experiments can be found in subrepository 3.

### Analyses Without Pandemic Time Constraints

Eliminating the 24-h runtime restriction, we also performed 3 *de novo* iterative experiments on both real and simulated data. In these Real and Simulated Unrestricted Experiments, we tested UShER + matOptimize, ML IQ-TREE 2 with stochastic search, and RAxML-NG on iterations of ~4.5k, ~8.9k, and ~13.2k samples, allowing each to run for up to 14 days. For runs that did not terminate within this time (the 2nd and 3rd iterations of RAxML-NG), we used the best tree inferred during the run for comparisons. We ran IQ-TREE 2 without the “-n 1” parameter as in previous experiments, enabling stochastic search. We ran IQ-TREE 2 and RAxML-NG under the GTR+G model with the smallest minimum branch length parameter that did not cause numerical errors. To compare the trees in the Real Unrestricted Experiments, we computed log-likelihoods under the GTR+G model for all trees, fixing the model parameters to those estimated by IQ-TREE 2 during tree inference. We also compared the log-likelihoods of the trees under the parameters estimated by RAxML-NG for the 1st iteration, but could not do so for the 2nd and 3rd iterations which did not terminate in under 2 weeks. We allowed optimization of branch lengths during likelihood calculation. For the UShER+matOptimize trees, before computing likelihoods, we converted the branch lengths into units of substitutions per site by dividing each branch length by the alignment length (29,903). To compare the trees inferred in the Simulated Unrestricted Experiments, we computed the RF distance and quartet similarity of each tree to the corresponding ground truth tree described above.

### Correlation of Parsimony and Likelihood on Large SARS-CoV-2 Phylogenies

We also performed an experiment on the starting tree of 364,427 samples to measure the correlation between the 2 optimality metrics used in our main experiments, parsimony and JC likelihood. In our Correlation Optimization Experiment, we performed 6 rounds of FastTree 2, measured the parsimony score and JC likelihood of the resulting trees, and computed the Pearson correlation coefficient between parsimony and likelihood.

## Results and Discussion

### Online Phylogenetics is an Alternative to *de novo* Phylogenetics for Ongoing Studies

**Figure 1.**
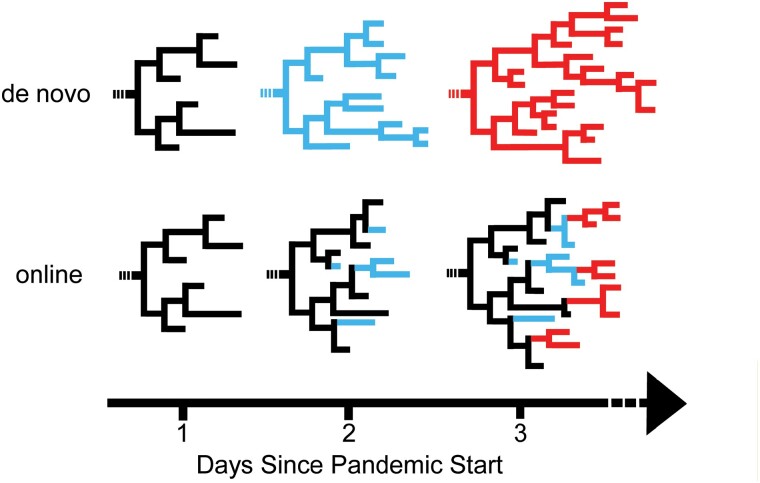
Phylogenies may be optimized from scratch using *de novo* phylogenetics or iteratively using online phylogenetics. In *de novo* phylogenetics (top), trees are repeatedly re-inferred from scratch. Conversely, online phylogenetics (bottom) involves placement of new samples as they are collected. Online methods may be particularly well suited to pathogen datasets like SARS-CoV-2 where close relatives of ancestral samples already exist in the phylogeny. Intermittent optimization steps (not depicted) after new samples are placed can help overcome errors from previous iterations. Online phylogenetics is expected to be much faster and require less memory than *de novo* phylogenetics.

The vast majority of phylogenetics during the pandemic has consisted of *de novo* phylogenetics approaches (Hadfield et al. 2018; Li et al. 2020; Lu et al. 2020a, 2020b; Meredith et al. 2020), in which each phylogeny is inferred using only genetic variation data, and without a guide tree (Fig. 1). This strategy for phylogenetic inference has long been the default, as in most instances in the past, data are collected just once for a project, and more relevant data are rarely going to be made available in the near future. This process is well characterized and has been foundational for many phylogenetics studies (Hug et al. 2016; Parks et al. 2018; Lu et al. 2020b), and most phylogenetics software is developed with *de novo* phylogenetics as the primary intended usage.

A challenging aspect of pandemic phylogenetics is the need to keep up with the pace of data generation as genome sequences continuously become available. To evaluate phylogenetics applications in the pandemic (Fig. 1), we split 233,326 samples dated from 23 December 2019 through 11 January 2021 into 50 batches according to their date of collection. Each batch contains roughly 5000 samples. Samples in each batch were collected within a few days of each other, except in the 1st months of the pandemic when sample collection was more sparse. We also constructed a data set of otherwise similar data simulated from a known phylogeny (see Methods). The intent of this scheme is to roughly approximate the data generation and deposition that occurred during the pandemic. All data sets are available from the repository associated with this project (Thornlow et al. 2021b), for reproducibility and so that future methods developers can directly compare their outputs to our results. We performed online and *de novo* phylogenetics using a range of inference and optimization approaches. Since thousands of new sequences are added to public sequence repositories each day, we terminated any phylogenetic inference approaches that took more than 24 h, because such phylogenies would be obsolete for some public health applications by the time they were inferred.

### Analyses Using Simulated Data Suggest that Online Phylogenetics is More Accurate for SARS-CoV-2

We first compared matOptimize (conda version 0.5.0) (Ye et al. 2022), ML IQ-TREE 2 (Minh et al. 2020), and FastTree 2 (Price et al. 2010) using both online and *de novo* phylogenetics strategies using simulated data that we designed to closely mimic real SARS-CoV-2 data sets. All Simulated Online Experiments used UShER (Turakhia et al. 2021a) to add new sequences to the previous tree (see Methods) as to the best of our knowledge it is the only software package that is fast enough to perform under real-time constraints. We chose these 3 tools to cover several different approaches and based on their widespread usage among SARS-CoV-2 phylogenetics applications. For example, matOptimize is part of the UShER suite (Turakhia et al. 2021a), IQ-TREE 2 is used by ([Bibr CIT0008][Bibr CIT0031]) and FastTree 2 is used by (Hadfield et al. 2018).

Simulating an alignment based on a known tree ensures that there is a ground truth for comparison to definitively assess each optimization method. We used an inferred global phylogeny as a template to simulate a complete multiple sequence alignment using phastSim (De Maio et al. 2022). We subsampled this simulated alignment into 50 progressively larger sets of samples, ranging in number of samples from 4676 to 233,326, to examine each of the 3 optimization methods in both online and *de novo* phylogenetics. For each iteration, we condensed identical samples, collapsed very short branches, and computed the RF distance to the condensed, collapsed ground truth global tree on which the simulation was based (see Methods). We also computed a quartet similarity metric (Asher and Smith 2022) to the ground truth tree. The ground truth tree was pruned to contain only the relevant samples for each iteration.

**Figure 2.**
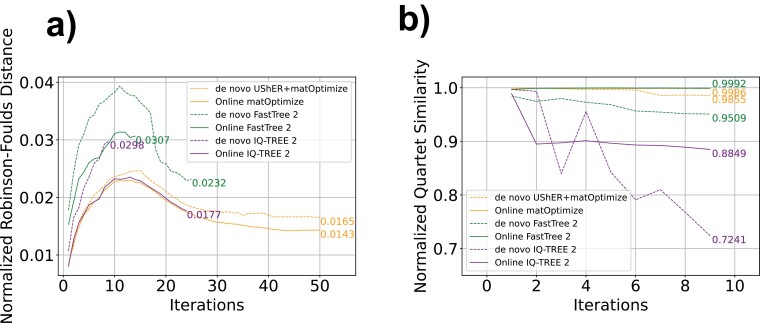
Online matOptimize produces highly similar phylogenies to ground truth on simulated data. For each batch of samples in our Simulated Online and *De Novo* Experiments, we calculated the normalized Robinson–Foulds (RF) distance between the tree produced by a given optimization software and the ground truth tree pruned to contain only the relevant samples, first collapsing near-zero branch lengths into multifurcations in both trees. To normalize RF distances, we divided each distance by $I(T_{i})\;+\;N_{i}-3$, where $I(T_{i})\;\;\;$ is the number of internal edges in the pruned ground truth tree for iteration i, and $N_{i}$ is the number of taxa (see Supplementary Fig. S1). We computed a quartet similarity metric to the ground truth tree for ten iterations of each method, normalizing by the maximum possible score of the metric as described in (Asher and Smith 2022). We terminated FastTree and IQ-TREE 2 after the first phylogeny that took more than 24 h to optimize.

All Simulated Online Experiments noticeably outperformed their *de novo* counterparts in the majority of iterations. Overall, online matOptimize produced phylogenies with the lowest normalized RF distance to the ground truth for the majority of iterations (Fig. 2a). Online IQ-TREE 2 performed similarly in RF distance but was able to complete only 24 of the 50 iterations due to its extreme computational resource requirements. When measured by quartet similarity, FastTree 2 produced trees closest to ground truth for the majority of the 14 iterations it completed (Fig. 2b). *De novo* IQ-TREE 2 performs relatively worse when measured by quartet similarity, perhaps due to errors early in tree inference that significantly affect the quartet similarity. For example, for the 9th phylogeny of 39,621 sequences, which was the last phylogeny produced using under 200 GB of RAM in under 24 h by all 6 methods, we found normalized RF distances of 0.0232, 0.0376, and 0.0297 for *de novo* UShER*+*matOptimize, FastTree 2, and IQ-TREE 2, respectively, and distances of 0.0220, 0.0296, and 0.0225 for online matOptimize, FastTree 2, and IQ-TREE 2, respectively. The distance of all methods to the ground truth tree are exceptionally small, possibly due to the “near perfect” phylogenetic properties of SARS-CoV-2 data (Wertheim et al. 2022).

There are many possible explanations for the improved performance of online phylogenetics on simulated data relative to *de novo* approaches. One possibility is that smaller trees are easier to optimize, so online phylogenetics will tend to estimate accurate initial trees to which later samples are added (this occurs because the radius for SPR moves when optimizing a large tree is too small to find improvements that are more readily applied when the tree contains fewer samples). In online phylogenetics, these improvements carry over to subsequent trees, while in *de novo* phylogenetics, they do not. The radius is defined as the phylogenetic distance of the search space when moving a node to a more optimal position. As the phylogeny increases in size, the distance from a node to its optimal position is likely to also increase, necessitating a larger SPR move radius to make equivalent improvements in larger trees. Additionally, the temporal nature of sample placement likely aids the performance of online approaches, with newer samples added to the tree after older samples.

### Analyses Using Real Data Suggest that Online Phylogenetics is More Efficient than *de novo* and Produces Similarly Optimal Phylogenies

**Figure 3.**
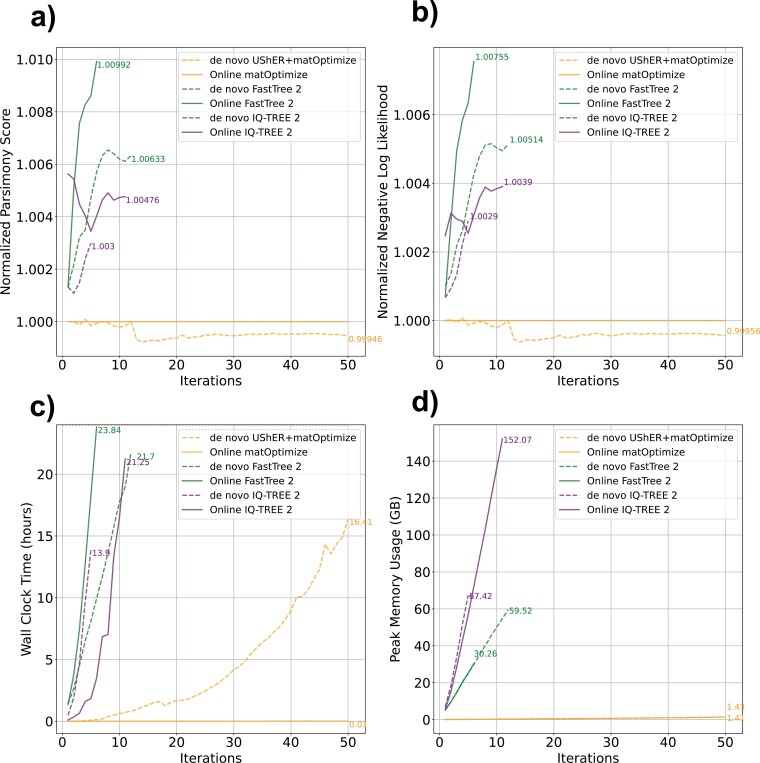
In practice, optimization by parsimony with matOptimize is more effective for SARS-CoV-2 data than optimization by established ML or pseudo-ML methods. For each Real Online and *De Novo* Experiment, we calculated (A) the parsimony score of each tree using matUtils, (B) the log-likelihood of each tree using IQ-TREE 2, (C) runtime and (D) peak memory usage of each optimization. (A) and (B) are normalized by the value obtained for the matOptimize online approach such that all other methods are expressed as a ratio. Strategies that surpassed 24 h (C) or the allowable RAM usage (D) were terminated prior. In most cases, with the notable exception of FastTree 2, online phylogenetics (solid lines) perform better than *de novo* phylogenetics (dashed lines). We ran all matOptimize analyses using an instance with 15 CPUs and 117.2 GB of RAM, and we ran all IQ-TREE 2 and FastTree 2 analyses on an instance with 31 CPUs and 244.1 GB of RAM, but limited each command to 15 threads for equivalence with matOptimize.

While analyses using simulated data offer the ability to compare to a known ground truth, assessing the performance of each method on real SARS-CoV-2 data may more accurately reflect practical use of each method. Therefore, we also tested each optimization strategy on 50 progressively larger sets of real SARS-CoV-2 samples in our Real Online and *De Novo* Experiments and calculated the parsimony score and likelihood of each optimized tree, as well as the run-time and peak RAM usage of each software package used (Fig. 3). To accomplish this, we subsampled our global phylogeny, which was produced using stringent quality control steps (see Methods), as before, to mimic the continuous accumulation of samples over the course of the pandemic.

Online optimizations are generally much faster than *de novo* phylogenetic inference. For example, IQ-TREE 2 achieves a roughly 7-fold faster run-time for online optimizations compared to inferring the tree *de novo* (Fig. 3c). The 5th iteration, which has 22,012 sequences and was the last to be completed by both online and *de novo* IQ-TREE 2, took 13 h 53 min for *de novo* IQ-TREE 2 but only 1 h 50 min for online IQ-TREE 2. *De novo* UShER+matOptimize was the only *de novo* method to finish all trees in fewer than 24 h, but its speed for each daily update pales in comparison to online matOptimize. Online matOptimize is several orders of magnitude faster than its *de novo* counterpart, and its optimizations for the largest phylogenies take roughly 30 s, while *de novo* tree inference with UShER can take several hours for trees consisting of more than 100,000 samples (Fig. 3c). However, whether a software package is used for online or *de novo* phylogenetics does not strongly affect its peak memory usage.

We also found that online phylogenetics strategies produce trees very similar in both parsimony score and likelihood to their *de novo* counterparts, with differences of less than 1% in all cases (Fig. 3a and b). For example, in the 5th iteration containing 22,012 sequences, online IQ-TREE 2 produces a tree with a parsimony score of 13,393, whereas *de novo* IQ-TREE 2 produces a tree with parsimony score of 13,387. Our results suggest that in addition to the computational savings that allow online phylogenetics approaches to continuously stay up-to-date, online phylogenetics approaches also produce trees with similar parsimony scores and likelihoods to their *de novo* counterparts.

### Under Pandemic Time Constraints, matOptimize has Equivalent or More Favorable Metrics Compared to ML and Pseudo-ML Methods for SARS-CoV-2 Phylogenies

In the case of both *de novo* and online phylogenetics, the parsimony-based matOptimize outperforms both FastTree 2 and IQ-TREE 2 in runtime and peak memory usage. For the 6th iteration (26,486 samples) of our Real Online and *De Novo* Experiments, which was the largest phylogeny inferred by all online methods in under 24 h and using under 200 GB of RAM, online FastTree 2 required nearly 24 h and 30.3 GB of RAM, and online IQ-TREE 2 required 3 h 29 min and 72 GB of RAM. By contrast, matOptimize required only 6 s and 0.15 GB of RAM. This iteration contained roughly 10% as many samples as the 50th and final iteration (233,326 total samples), which online matOptimize completed in 32 s using 1.41 GB of RAM at peak usage. Even this largest tree represents only a very small fraction of the more than 14 million currently available SARS-CoV-2 genomes, indicating that, among the approaches we evaluated, matOptimize is the only viable option for maintaining a comprehensive SARS-CoV-2 phylogeny via online phylogenetics.

In addition to its scalability, matOptimize outperformed ML and pseudo-ML optimization methods under 24-h time constraints in both the parsimony and likelihood scores of the trees that it inferred. For the 6th iteration (26,486 samples) of our Real Online Experiments, we found parsimony scores of 16,130, 16,195, and 16,290 for matOptimize, IQ-TREE 2, and FastTree 2, respectively. While all methods produced phylogenies with parsimony scores within 1% of each other, matOptimize was consistently the lowest. However, matOptimize was developed to optimize by parsimony, while the other methods optimize for likelihood. Unexpectedly, we found that the trees from matOptimize had better log-likelihood than those from likelihood-based software implementations. For the 6th iteration (26,486 samples) of our Real Online Experiments, we found log-likelihood scores of –233,414.277, –234,130.859, and –235,177.396 for matOptimize, IQ-TREE 2, and FastTree 2, respectively. IQ-TREE 2 and FastTree 2 inferred trees using a Generalized Time Reversible (GTR) model, but due to time constraints in calculation we used a JC model to compare likelihoods across methods. However, a GTR model with specified rate parameters produced strongly correlated likelihoods (Supplementary Table S1, Dryad: https://doi.org/10.7291/D13Q2J) (Thornlow et al. 2022). Indeed, we were able to calculate likelihoods under the GTR model for the first 5 iterations of the Real *De Novo* Experiments (from 4676 to 22,012 samples) for both the matOptimize and IQ-TREE 2 trees. In every case, the tree from matOptimize had a slightly better likelihood score than the tree from IQ-TREE 2.

### Parsimony Optimization with matOptimize Produces Comparable SARS-CoV-2 Trees to the Most Thorough ML Methods

**Figure 4.**
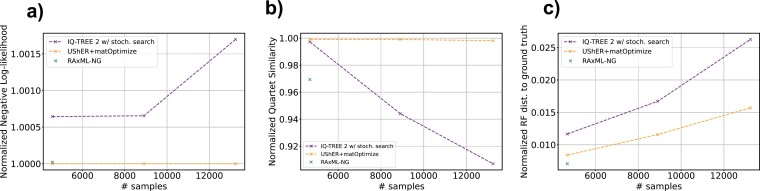
*De novo* matOptimize produces comparable trees to the most thorough ML inference programs. We ran these methods for up to 2 weeks each to infer trees *de novo* from the 3 smallest iterations of real and simulated data. For real data (A), log-likelihoods were computed under the model parameters estimated by IQ-TREE 2 at each iteration. Values are normalized by the value of the matOptimize approach such that all other methods are expressed as a ratio. For simulated data (B, C), the reported quartet similarities are normalized by the maximum value of the metric described in (Asher and Smith 2022). RF distances (C) are normalized by dividing by    I(Ti) + Ni − 3, where I(Ti)  is the number of internal edges in the pruned ground truth tree for iteration i, and $N_{i}$ is the number of taxa. For all panels, the 2nd and 3rd iterations of RAxML-NG (which did not terminate within 2 weeks) are omitted.

We also compared the performance of *de novo* inference with UShER+matOptimize to state-of-the-art methods without a 24-h limit on runtime in our Real and Simulated Unrestricted Experiments. In 3 iterations of increasing size (~4.5k, ~8.9k, and ~13.2k samples), we inferred trees from real and simulated data using UShER+matOptimize, IQ-TREE 2 with stochastic search enabled, and RAxML-NG. We allowed each experiment to run for up to 2 weeks. All programs completed successfully on the 1st iteration. RAxML-NG did not terminate within 2 weeks for the 2nd and 3rd iterations. On real data, we found that UShER+matOptimize produced trees with higher log-likelihoods than IQ-TREE 2 and RAxML-NG across all 3 iterations (Fig. 4a). Under the substitution model parameters estimated by IQ-TREE 2, the log-likelihoods for the 1st iteration were –73,780.756, –73,828.271, and –73,782.289 for UShER+matOptimize, IQ-TREE 2, and RAxML-NG, respectively. Under the parameters estimated by RAxML-NG, the log-likelihoods for the 1st iteration were –––––73,754.894, –73,801.935, and –73,756.246 for UShER+matOptimize, IQ-TREE 2, and RAxML-NG, respectively. On simulated data, UShER+matOptimize produced trees closer to the ground truth than the other methods when measured by quartet similarity across all 3 iterations (Fig. 4b). By RF distance, the UShER+matOptimize trees were closest to the ground truth for the 2nd and 3rd iterations, but the RAxML-NG tree was closest to ground truth in the 1st iteration (Fig. 4c). On the 1st iteration of simulated data, we found normalized RF distances of 0.0084, 0.0117, and 0.0071 for UShER+matOptimize, IQ-TREE 2, and RAxML-NG, respectively. We found normalized quartet similarities of 0.999, 0.997, and 0.969 for UShER+matOptimize, IQ-TREE 2, and RAxML-NG, respectively. We therefore conclude that parsimony-based tree inference with matOptimize can perform equivalently to state of the art ML approaches and can do this in a tiny fraction of the time, making it by far the most suitable approach for pandemic-scale phylogenetics of SARS-CoV-2.

### Parsimony and Likelihood are Strongly Correlated When Optimizing Large SARS-CoV-2 Phylogenies

**Figure 5.**
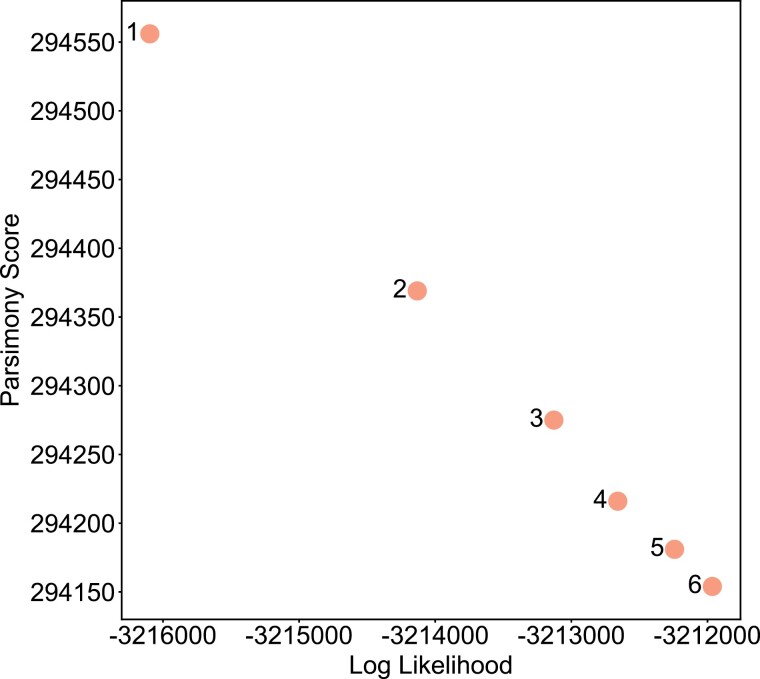
Improvement in likelihood and parsimony have a linear relationship for our optimized global tree. We optimized our initial global tree using 6 iterations of FastTree and measured the total parsimony and the likelihood after each, finding a linear relationship (Pearson correlation, rho = –1.0, *P* < 2.9 × 10^–7^).

In our Correlation Optimization Experiment on the starting tree of 364,427 SARS-CoV-2 genomes, we found that after each of 6 iterations of FastTree 2 optimization, the JC likelihood and parsimony improvements are strongly linearly correlated (Fig. 5). This suggests that changes achieved by maximizing parsimony will also optimize likelihood for SARS-CoV-2 data. That is, for extremely densely sampled phylogenies in which long branches are rare, parsimony and likelihood are highly correlated, as are the effects of tree moves to optimize either. However, despite the strength of this correlation, we find an extreme disparity in practical usage when optimizing by either metric. Parsimony-based methods are far more time- and data-efficient, and presently available ML and pseudo-ML approaches quickly become prohibitively computationally expensive. For example, while the 6 iterations of FastTree did result in large improvements in both likelihood and parsimony score, the resulting tree would be out of date long before the 10.5-day optimization had completed. Moreover, we applied matOptimize to the tree output by the 6th iteration of FastTree, achieving a parsimony score of 293,866 (improvement of 288) and a JC log-likelihood of -3483329.485 (improvement of 2318.535) in just 16 min, indicating that even after 10.5 days, additional optimization was still possible. This suggests that, for the purposes of optimizing even moderately large SARS-CoV-2 trees, parsimony-based methods should be heavily favored due to their increased efficiency.

## Conclusions

The SARS-CoV-2 pandemic has made phylogenetics central to efforts to combat the spread of the virus, but has posed challenges for many commonly used phylogenetics frameworks. A major component of this effort relies on a comprehensive, up-to-date, global phylogeny of SARS-CoV-2 genomes. However, the scale and continuous growth of the data have caused difficulties for standard *de novo* phylogenetic methods. Here, we find that online phylogenetics methods are practical, pragmatic, and accurate for inferring daily phylogenetic trees from a large and densely sample virus ountbreak.

One counterintuitive result is that parsimony-based optimization outperformed sufficiently efficient ML and pseudo-ML approaches in the majority of our experiments regardless of whether phylogenies were evaluated using parsimony or likelihood. This might be a consequence of the fact that parsimony scores and likelihoods are strongly correlated across phylogenies inferred via a range of phylogenetic approaches. The extremely short branches (Supplementary Fig. S2) on SARS-CoV-2 phylogenies mean that the probability of multiple mutations occurring at the same site on a single branch is negligible. Stated another way, SARS-CoV-2 is approaching a “limit” where parsimony and likelihood are nearly equivalent. Similar observations have been made in other fields that, in certain cases, nonparametric methods based on parsimonious solutions perform similarly to statistical methods that assume an underlying model (Huelsenbeck et al. 2000; Ree et al. 2005; Nylander et al. 2008). In turn, because of its relative efficiency, matOptimize is able to search more of the possible tree space in the same amount of time, thereby resulting in trees with better likelihoods and lower parsimony scores than trees optimized using currently available ML software packages. We emphasize that this does not bear on the relative merits of the underlying principles of ML and MP, but instead reflects the utility of methods that have been applied during the pandemic. Nevertheless, this observation does suggest that in some cases, MP optimization may provide a fast and accurate starting point for ML optimization methods. Indeed, many popular phylogenetic software packages such as RAxML (Stamatakis 2014) and IQ-TREE (Minh et al. 2020) already use stepwise-addition parsimony trees as initial trees for their optimization or in conjunction with likelihood during inference as in PhyML 3.0 (Guindon et al. 2010). Our results suggest that further optimization of initial trees using MP may provide benefits in speed *and* accuracy for some data sets, even when the target is an estimate of the ML tree.

As sequencing technologies progress, sample sizes for phylogenetic analyses of major pathogens and highly studied organisms will necessarily continue to increase. Today, SARS-CoV-2 represents an extreme with respect to the total number of samples relative to the very short branch lengths on the phylogeny. However, the global sequencing effort during the pandemic suggests that the public health sphere has a strong interest in the increased application of whole-genome sequencing to study the genomic contents, evolution, and transmission history of major and emerging human pathogens. In addition to our global tree of SARS-CoV-2 with ~14 million samples, we use online phylogenetics with UShER and matOptimize to maintain 3 other publicly available viral phylogenies, with a web server for sample placement hosted at https://genome.ucsc.edu/cgi-bin/hgPhyloPlace. As of February 2023, our Mpox tree contains 3751 genomes, our RSV-A tree contains 2592 genomes, and our RSV-B tree contains 1915 genomes. In addition, we have applied UShER and matOptimize to the bacterial pathogen Mycobacterium tuberculosis, constructing a *de novo* tree with 10,248 genomes (Ye et al. 2022). All of these pathogen trees share relatively dense sampling and short average branch lengths, so the results of this paper suggest they are well-suited to inference using online parsimony methods. We expect that million-sample data sets will become commonplace in the near future, and that parsimony-based methods like matOptimize show promise for many pathogens beyond SARS-CoV-2. Recently developed parsimony-based likelihood approximations may ultimately be similarly scalable and accurate (De Maio et al. 2023). Online phylogenetics using both of these methods will be a fruitful avenue for future development and application to accommodate these data sets.

## SUPPLEMENTARY MATERIAL

Data available from the Dryad Digital Repository: https://doi.org/10.7291/D13Q2J.

## Data Availability

The data underlying this article are available online at https://hgwdev.gi.ucsc.edu/~angie/, and can be accessed at the following file identifiers: publicMsa/publicMsa.2021-03-18.masked.pb, publicMsa/publicMsa.2021-03-18.masked.vcf.xz, publicMsa/publicMsa.2021-03-18.masked.fa.xz, publicMsa/publicMsa.2021-03-18.nwk, and UShER_SARS-CoV-2/2021/03/18/public-2021-03-18.metadata.tsv.gz. The UShER web server is hosted at https://genome.ucsc.edu/cgi-bin/hgPhyloPlace. UShER and matOptimize are available to download via Anaconda at https://anaconda.org/bioconda/usher and on Github at https://github.com/yatisht/usher. All data analysis scripts and additional data are available on Github at https://github.com/bpt26/parsimony and the Dryad Digital Repository: https://doi.org/10.7291/D13Q2J. Supplemental figures are available on Dryad.
